# Back to Basics: A Case of Adult Epiglottitis

**DOI:** 10.7759/cureus.3475

**Published:** 2018-10-22

**Authors:** Sidhartha R Ramlatchan, Nicholas Kramer, Latha Ganti

**Affiliations:** 1 Emergency Medicine, Osceola Regional Medical Center, Kissimmee, USA; 2 Emergency Medicine, University of Central Florida College of Medicine, Orlando, USA

**Keywords:** epiglottitis

## Abstract

The authors report a case of a 69-year-old female with difficulty swallowing and neck swelling and review the clinical presentation, radiographic features and treatment of adult epiglottitis. Epiglottitis remains a medical emergency, with the potential for airway compromise. The authors present this case because it is a potentially life-threatening infection that warrants prompt diagnosis and management. This case is worth reporting because epiglottis in adults can have a milder and less classic presentation that sometimes results in delayed recognition.

## Introduction

Acute epiglottitis, also known as supraglottitis, is an invasive cellulitis involving the epiglottis and the adjacent structures including the aryepiglottic folds and vallecula. Prior to widespread vaccination against haemophilus influenzae in the 1990s, it was primarily a bacterial illness seen in young children, with a peak age of three years [1]. The epidemiology has now shifted towards adults [2]. Epiglottitis in adults differs from that in children. It is typically a less severe, non-bacteremic infection that has a longer duration of symptoms, with a predominant complaint of sore throat [3].

The definitive diagnosis of epiglottitis is made by visualization of an edematous, cherry red epiglottis by direct or indirect laryngoscopy or fiberoptic endoscopy [4]. Lateral neck radiographs will demonstrate an enlarged epiglottis with ballooning of the hypopharynx and prevertebral soft tissue swelling [5,6].

Acute epiglottitis has the potential for causing abrupt, complete airway obstruction and is thus a medical emergency that requires prompt diagnosis and careful management of the airway. While less common in adults, it has more potential for poor outcome due to the often indolent nature of the clinical course. The authors present a case of acute epiglottitis in an adult.

## Case presentation

A 69-year-old female presented to our emergency department with chief complaint of neck swelling, sore throat and difficulty swallowing for the past two days. She also complained of fever and voice hoarseness. Flexion and extension of the neck made the pain worse. She denied difficulty breathing, nausea, vomiting, or chest pain. She reported a non-productive cough. She stated that her daughter and grandchildren were sick with a cough and cold. She took diphenhydramine, acetaminophen and some old amoxicillin at home. Her past medical history was significant for salivary gland cancer nine years prior that was treated with chemotherapy and radiation.

**Figure 1:**
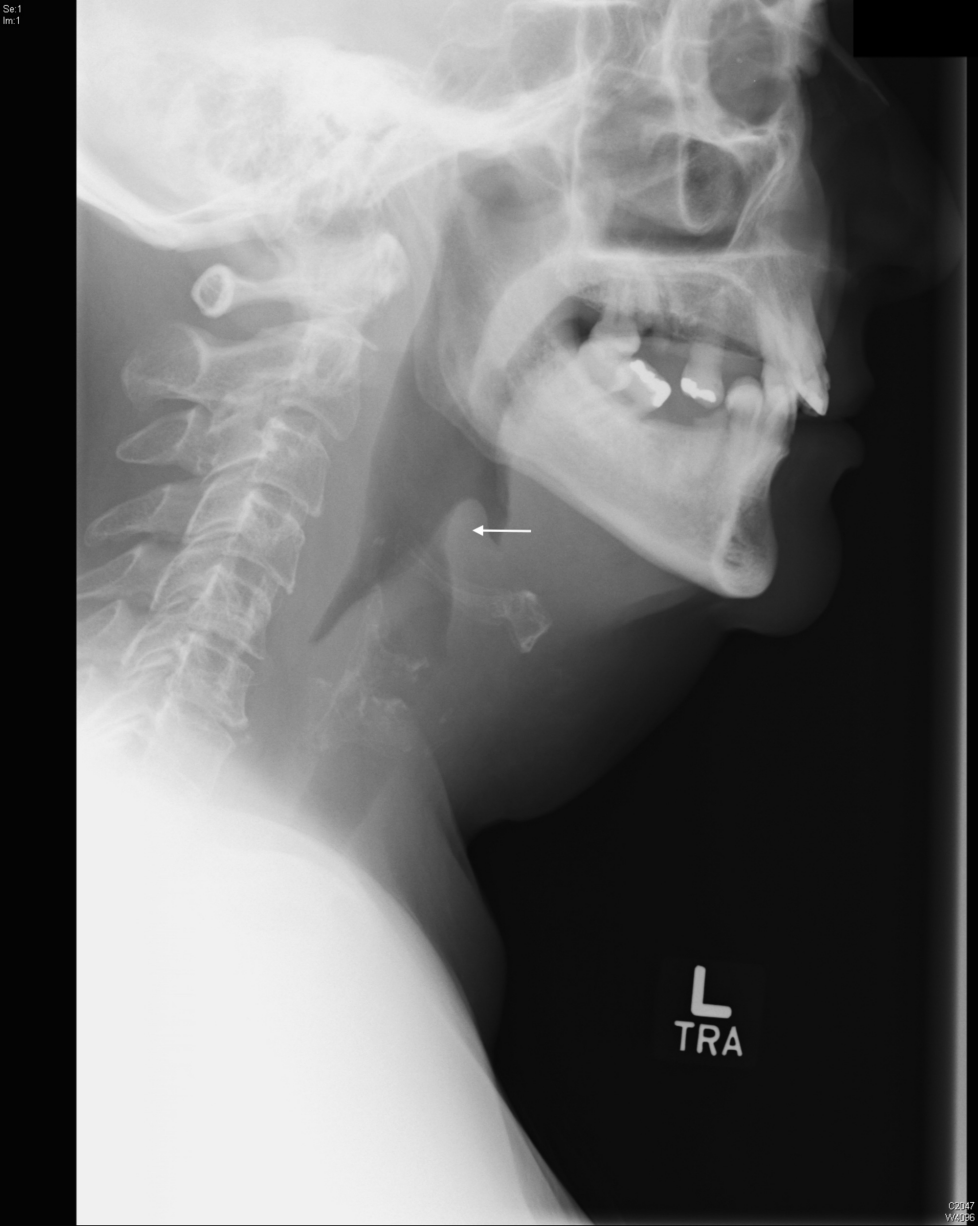
Radiograph demonstrating enlarged epiglottis and aryepiglottic folds.

Her vital signs were: pulse 103, blood pressure 120/62 mmHg, temperature 37.6°C, respirations 18/min, and room air pulse oximetry 100%. Laboratory analyses revealed an elevated white blood cell count of 33 K/mm^3^, and an elevated procalcitonin level of 23.67 ng/mL. Radiograph of the soft tissues of the neck revealed an enlarged epiglottis and aryepiglottic folds, consistent with epiglottitis. In addition, subglottic narrowing of the airway is demonstrated (Figure 1). The patient received intravenous ampicillin-sulbactam, methylprednisolone and diphenhydramine in the emergency department with some improvement. The patient was initially admitted to the intensive care unit and evaluated by the otolaryngology service using a flexible nasopharyngoscope. The patient was gradually weaned off of intravenous medications and discharged on hospital day three. Blood and throat cultures were negative.

## Discussion

Acute epiglottitis is a disease in which the epiglottis is inflamed, obstructing a patient’s respiratory system [1]. The epiglottis is responsible for regulating air into the lungs, and is crucial to the performance of a patient’s upper airways. Epiglottitis in adults is not as commonly associated with bacteremia as in children. If a bacterial isolate is identified, the more common pathogens are Streptococcus pneumoniae, Streptococcus pyogenes, Neisseria meningitidis or Group A b-hemolytic Streptococcus [2]. Haemophilus influenzae B (HIB) is also an important pathogen in adult epiglottitis. This is now rarely seen in children, since widespread vaccination started two decades ago. However, the etiology may also be nonbacterial [7]. Trauma of the epiglottis can be induced by foreign objects, chemical burns, or reactions to chemotherapy [2]. The epiglottis gets smaller and more rigid once a patient reaches adulthood, making epiglottitis less severe in adults due to lesser clogging of the airway by the inflammation [3].

The clinical presentation of epiglottitis begins like many upper respiratory infections, with sore throat, fever, and malaise. Due to these minor initial symptoms, acute epiglottitis is often overlooked in adults, allowing the disease to unknowingly progress. While the progression occurs rapidly over a span of hours for children, an adult’s expanded upper airway allows the disease to progress over a span of days. The most common initial symptom is a minor sore throat that increases in severity over time, which may be accompanied with a voice change or cough. Over time the patient may develop a variety of symptoms affecting their respiratory system, the more common ones being odynophagia, an inability to swallow secretions, and dyspnea [8]. The “thumbprint sign” is a reliable predictor of rapid airway obstruction seen on lateral X-ray of the patient’s neck [9], and is noted up to 88% of the time [10,11], with the width of the epiglottis in adults exceeding 8 mm, and the width of the aryepiglottic folds exceeding 7 mm [12]. Direct examination of the oropharynx as an initial step generally is safer in adults than in children [13], but lateral neck films are usually obtained early in the diagnostic evaluation.

Patients experiencing upper airway obstruction as a result of acute epiglottitis must be treated to have their airways opened, and allow air to flow through their respiratory system [14]. Treatment generally consists of intravenous antibiotics, hydration with normal saline and corticosteroids to decrease local inflammation. Patients should be admitted to the intensive care unit after securing the airway [8].

Differential diagnosis includes bacterial tracheitis, thermal epiglottitis (scald burn from smoke or hot beverages), possibly angioneurotic edema, retropharyngeal or peritonsillar abscesses, uvulitis, and diphtheria.

Complications include epiglottic abscess and systemic bacteremia.

## Conclusions

In this case report the authors present a case of epiglottitis. If left untreated, epiglottitis can progress to life-threatening airway obstruction, and thus remains a diagnosis not to be missed.
